# Prognostic Value of Parathyroid Hormone in Heart Failure with Reduced Ejection Fraction

**DOI:** 10.3390/jcm15134859

**Published:** 2026-06-23

**Authors:** Ahmet Genç, Gülsüm Meral Yılmaz Öztekin, Şükriye Uslu, Rauf Avcı

**Affiliations:** Department of Cardiology, Antalya Training and Research Hospital, Antalya 07100, Türkiye; gulsummeral.yilmaz@sbu.edu.tr (G.M.Y.Ö.); sukriyeuslu@gmail.com (Ş.U.); drraufavci07@gmail.com (R.A.)

**Keywords:** heart failure, parathyroid hormone, NT-proBNP, prognosis, biomarkers, mortality

## Abstract

**Background/Objectives**: Parathyroid hormone (PTH) has emerged as a novel biomarker in heart failure (HF), reflecting neurohormonal, renal, and metabolic dysregulation within the cardiorenal–mineral axis. However, its independent prognostic value and incremental contribution remain unclear when evaluated through formal nested structures Therefore, this study aimed to evaluate the association between PTH and all-cause mortality in patients with heart failure with reduced ejection fraction (HFrEF) and to determine whether PTH provides additional prognostic information beyond NT-proBNP. **Methods**: In this retrospective cohort study, 1594 patients with HFrEF (LVEF ≤ 40%) were analyzed. Serum PTH and NT-proBNP levels were log-transformed and evaluated as predictors of all-cause mortality. Patients were stratified according to PTH levels, and survival analysis was performed. Incremental model fit was evaluated using nested likelihood ratio tests. Stratified multivariable Cox models and formal interaction tests were executed across predefined clinical strata (age, renal function, and heart failure etiology). **Results**: During a median follow-up of 36 months, 525 deaths occurred. Elevated PTH levels were associated with worse survival outcomes. In multivariable Cox regression analysis, both LnPTH (HR: 1.233, *p* = 0.0147) and LnNT-proBNP (HR: 1.374, *p* < 0.0001) were independent predictors of mortality. Combined elevation of PTH and NT-proBNP identified patients at the highest risk. The addition of LnPTH to the baseline model significantly improved global model fit (χ^2^ = 4.242, *p* = 0.0394). Importantly, the prognostic value of LnPTH was significantly modified by age (*P_interaction_* = 0.026) and renal function (*P_interaction_* = 0.038), demonstrating independent predictive power specifically in patients aged < 65 years (HR: 1.402) and those with eGFR ≥ 60 mL/min/1.73 m^2^ (HR: 1.454), but not in older or advanced renal impairment strata. **Conclusions**: PTH is independently associated with mortality in patients with HFrEF and provides incremental prognostic value beyond NT-proBNP by optimizing global model fit. These findings support its role as a complementary biomarker within a multimarker strategy for improved risk stratification of the cumulative metabolic and cardiovascular burden.

## 1. Introduction

In heart failure (HF), parathyroid hormone (PTH)—traditionally regarded as a regulator of calcium–phosphate homeostasis—has emerged as a promising candidate with potential cardiovascular relevance [1]. The presence of PTH receptors in cardiomyocytes, vascular smooth muscle cells, and endothelial cells indicates that PTH may exert direct cardiovascular effects beyond its classical endocrine functions [2].

Experimental studies have demonstrated that elevated PTH levels can induce cardiomyocyte hypertrophy, promote intracellular calcium overload, impair mitochondrial function, and increase oxidative stress, ultimately leading to myocardial fibrosis and cellular injury [3,4,5]. These structural and functional alterations contribute to adverse ventricular remodeling, increased arrhythmogenicity, and progressive HF [6]. Moreover, PTH has been implicated in vascular and valvular calcification, arterial stiffness, and endothelial dysfunction, pathways known to exacerbate HF severity [3,7]. The interaction between PTH and the renin–angiotensin–aldosterone system (RAAS) further amplifies neurohormonal activation, creating a deleterious cycle that accelerates HF progression [8].

Clinical studies have consistently shown that elevated PTH levels are associated with worse outcomes in HF, independent of serum calcium and phosphate levels, vitamin D status, renal function, and established cardiovascular risk factors [9]. Even within the normal reference range, higher PTH concentrations have been linked to increased cardiovascular mortality in community-based populations [10]. In patients with HF, PTH has been associated with all-cause mortality, rehospitalization, and advanced disease severity, independent of left ventricular ejection fraction (LVEF) and other conventional prognostic markers [11,12,13]. These findings suggest that PTH may serve as a multidimensional biomarker reflecting cardiac stress, neurohormonal activation, and systemic metabolic dysregulation.

Importantly, recent evidence indicates that PTH may complement natriuretic peptides in prognostic assessment. The combination of PTH and NT-proBNP has been shown to improve risk prediction for mortality and HF-related hospitalizations, suggesting that these biomarkers capture distinct yet synergistic aspects of HF pathophysiology [1,14,15]. This raises the possibility that PTH could enhance current biomarker-based strategies, particularly in patients with reduced ejection fraction (HFrEF), where accurate risk stratification is essential for guiding therapeutic decisions.

Despite these promising findings, most existing studies have been limited by relatively small sample sizes, short follow-up durations, or heterogeneous patient populations. Consequently, the independent prognostic value of PTH, especially its incremental contribution beyond NT-proBNP, remains insufficiently clarified in large, well-characterized HFrEF cohorts. Furthermore, there is a lack of robust evidence demonstrating whether PTH provides incremental prognostic value beyond traditional natriuretic peptides when evaluated through formal nested model structures.

Based on the current gaps in the literature, the present study was designed to investigate whether PTH serves as an independent risk factor for all-cause mortality in patients with HFrEF and to determine whether PTH provides additional prognostic value beyond NT-proBNP. Leveraging a large cohort of 1594 patients and long-term follow-up, this study aims to offer robust and comprehensive evidence regarding the clinical significance of PTH in the prognostic evaluation of HFrEF, evaluating global model fit via nested likelihood ratio tests to determine its clinical utility in refining risk stratification.

## 2. Methods

### 2.1. Study Design and Population

This retrospective, single-center cohort study was conducted at the Antalya Training and Research Hospital, Department of Cardiology. The study protocol was approved by the local ethics committee and conducted in accordance with the ethical principles of the Declaration of Helsinki.

**Figure 1:**
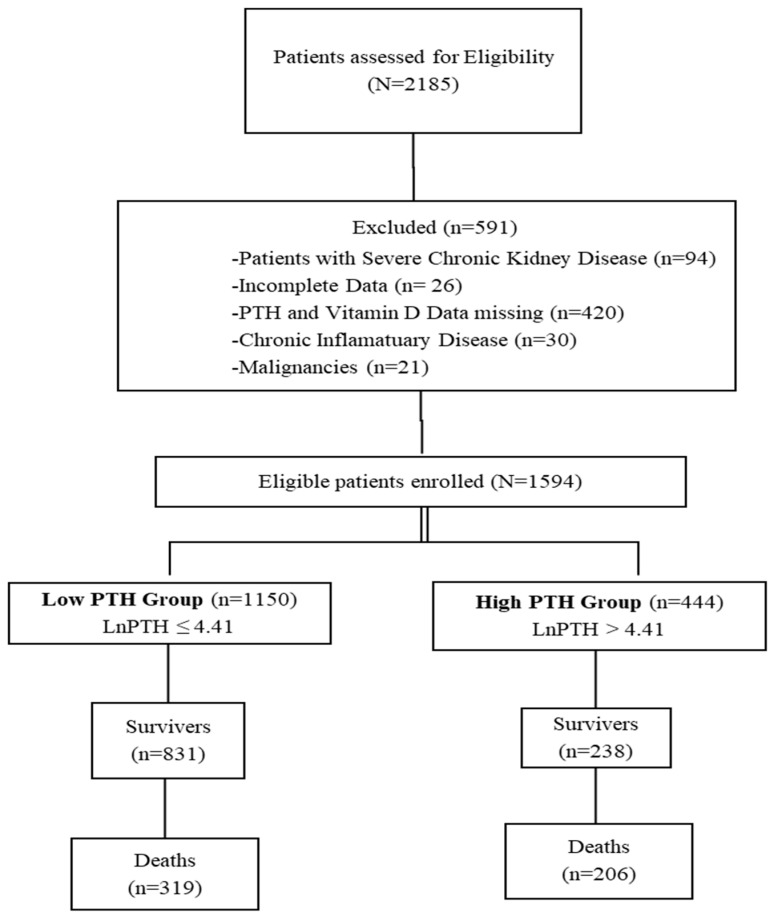
Study Flow Diagram of Patient Enrollment and Group Allocation. Flowchart summarizing patient screening, exclusion criteria, and final stratification into low and high PTH groups. Abbreviations: PTH, parathyroid hormone.

Between January 2014 and February 2026, a total of 2185 consecutive patients presenting to the HF outpatient clinic were screened. As illustrated in the Study Flow Diagram (Figure 1), the screening process is summarized below. The specific inclusion criteria were as follows: (1) age ≥ 18 years; (2) a documented diagnosis of heart failure with reduced ejection fraction (HFrEF) according to the European Society of Cardiology (ESC) guidelines, defined by a left ventricular ejection fraction (LVEF) ≤ 40% on index transthoracic echocardiography; and (3) stable clinical status for at least 3 months on guideline-directed medical therapy (GDMT). Exclusion criteria consisted of the following: (1) acute decompensated heart failure requiring intravenous inotropic or pressor support at baseline; (2) primary hyperparathyroidism or known intrinsic parathyroid malignancy; (3) patients with severe renal impairment (eGFR < 30 mL/min/1.73 m^2^); and (4) active malignancy or terminal non-cardiac disease with a life expectancy of less than 1 year. Of the 2185 screened patients, 420 were excluded due to missing baseline data, resulting in a final study cohort of 1594 patients. To address potential selection bias, a comprehensive sensitivity analysis was performed, which confirmed that the excluded individuals (*n* = 420) did not differ systematically from the final study cohort in terms of baseline clinical severity and NYHA functional class (all *p* > 0.05) (Supplementary Table S1).

### 2.2. Data Acquisition and Laboratory Measurements

Comprehensive clinical, demographic, and biochemical data were retrieved from the institutional electronic health record system. Baseline comorbidities and evidence-based medical therapies, including Angiotensin-Converting Enzyme inhibitors (ACEIs), Angiotensin II Receptor Blockers (ARBs), Angiotensin Receptor–Neprilysin Inhibitors (ARNIs), Beta Receptor Blockers (BB), Mineralocorticoid Receptor Antagonists (MRAs), Sodium–glucose co-transporter-2 inhibitors (SGLT2i), and diuretics, were meticulously recorded.

PTH and NT-proBNP levels were analyzed, and due to their non-normal distribution, the natural logarithm of these values (LnPTH and LnNT-proBNP) was used for all statistical analyses. Renal function was assessed using the Chronic Kidney Disease Epidemiology Collaboration (CKD-EPI) formula. Serum calcium levels were adjusted for albumin concentrations using the corrected calcium formula: [Total Calcium (mg/dL) + 0.8 × (4.0 − Serum Albumin (g/dL))]. 25(OH)D concentrations were determined for all participants.

As shown in Table 1, the study population was stratified into two distinct groups based on a data-derived optimal cut-off value of LnPTH = 4.41 (~82 pg/mL); the low-PTH group served as the internal clinical control reference population to evaluate the relative hazard of the high-PTH group. Serum 25(OH)D levels were measured to systematically account for and rule out secondary hyperparathyroidism due to severe vitamin D deficiency during multivariable risk adjustment. Transthoracic echocardiography data were retrieved from patient records, where LVEF had been calculated using the modified Simpson’s biplane method with a Philips EPIQ 7 system (Philips Healthcare, Andover, MA, USA).

### 2.3. Study Endpoints and Statistical Analysis

Statistical analyses were conducted using SAS software, version 9.4 (SAS Institute Inc., Cary, NC, USA). The distributional integrity of continuous variables was rigorously assessed through the Kolmogorov–Smirnov test and visual inspection of Q-Q plots. Data were presented as the mean ± standard deviation (SD) for normally distributed variables (|skewness| < 1) and as the median with interquartile range (IQR) for non-normally distributed data (|skewness| ≥ 1). Categorical variables were expressed as absolute frequencies and percentages. To stabilize variance and mitigate the effects of right-skewed distributions, natural logarithmic transformations (LnPTH and LnNT-proBNP) were applied to PTH and NT-proBNP values prior to their inclusion in the regression models.

The primary endpoint of the study was long-term all-cause mortality, defined as the time from the index outpatient visit to the date of death from any cause, with survival status tracked through the national electronic death registration system. Due to the retrospective nature of the study and the inherent limitations in the underlying electronic registry documentation, specific primary causes of death (such as cardiovascular versus non-cardiovascular specific etiologies) could not be systematically adjudicated. Consequently, long-term all-cause mortality was strictly maintained as the solitary hard clinical endpoint for all survival analyses to ensure maximal data integrity and prevent potential misclassification bias. Survival data for patients who did not reach the primary endpoint were censored at the date of their last clinical follow-up. Optimal prognostic cut-off values for LnPTH and LnNT-proBNP were identified via Receiver Operating Characteristic (ROC) curve analysis, utilizing the Youden index to determine thresholds that maximize the sum of sensitivity and specificity. Survival probabilities were estimated through the Kaplan–Meier method, and disparities between the stratified risk groups were statistically evaluated using the log-rank (Mantel–Cox) test. 

Independent predictors of mortality were identified through Cox proportional hazards regression models. To eliminate data-driven instability, residual confounding, and potential overfitting traditionally associated with automated step-by-step selection methods, independent predictors of mortality were evaluated using a pre-specified, clinically driven forced-entry multivariable Cox proportional hazards regression model. This model simultaneously and non-selectively adjusted for a robust set of clinically relevant factors including age, sex, LVEF, eGFR, hemoglobin, serum sodium, loop diuretic use, NYHA functional class, LnNT-proBNP, and key mineral–metabolic factors (corrected calcium and 25(OH)D), rather than allowing for automated exclusion during stepwise iterations. The incremental prognostic contribution of LnPTH beyond these conventional risk factors and LnNT-proBNP was formally quantified using the Net Reclassification Index (NRI) and Integrated Discrimination Improvement (IDI) at the 36-month timeline. The statistical significance of model optimization was rigorously tested by comparing global model fit between nested models (Baseline Model vs. Baseline Model + LnPTH) using the nested likelihood ratio test.

All statistical tests were two-tailed, and a *p*-value of <0.05 was considered to indicate statistical significance.

AI Usage Statement: During the preparation of this manuscript, the authors used Microsoft Copilot (2026 version) for assistance in language editing and structural refinement. The authors have reviewed and edited all AI-generated content and take full responsibility for the final version of the manuscript.

## 3. Results

### 3.1. Baseline Characteristics and Study Population

A total of 2185 patients with HFrEF were screened, and 1594 participants (mean age 62.1 ± 12.8 years; 73.3% male) were enrolled in the final analysis. As illustrated in the study flow diagram (Figure 1), a rigorous selection process was applied.

The cohort was stratified based on PTH levels into a low PTH group (LnPTH ≤ 4.41; ≤ ≈82 pg/mL; *n* = 1150) and a high PTH group (LnPTH > 4.41; >≈82 pg/mL; *n* = 444). Patients in the high PTH group were significantly older, had a longer HF duration (24 vs. 12 months), and presented with more advanced NYHA functional classes (*p* < 0.001). Laboratory analysis showed that elevated PTH levels correlated with lower eGFR (58 vs. 72 mL/min/1.73 m^2^), lower serum albumin (4.1 vs. 4.3 g/L), and higher NT-proBNP levels (2826 vs. 992 ng/L) (*p* < 0.001). Detailed baseline characteristics are provided in Table 1.

Regarding evidence-based medical therapies, a remarkable translational signal was observed in loop diuretic utilization patterns. Patients in the high PTH group exhibited a significantly higher prevalence of loop diuretic use compared to those in the low PTH group (58% vs. 79%, *p* < 0.001). Furthermore, the median daily dose of loop diuretics was substantially elevated in the high PTH cohort (20 mg/day [IQR: 0–40] vs. 40 mg/day [IQR: 20–40], *p* < 0.001).

### 3.2. Distribution of LnPTH Across NYHA Functional Classes

**Figure 2:**
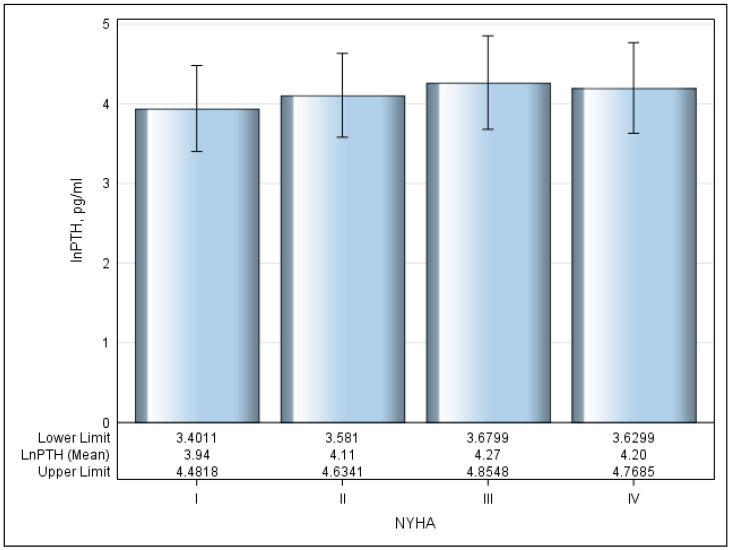
Distribution of Log-Transformed Parathyroid Hormone Levels Across NYHA Functional Classes. Serum PTH levels in relation to heart failure severity. The box-plot illustrates the progressive increase in log-transformed parathyroid hormone (LnPTH) concentrations across New York Heart Association (NYHA) functional classes (I to IV). Abbreviations: NYHA, New York Heart Association; LnPTH, log-transformed intact parathyroid hormone.

The relationship between secondary hyperparathyroidism and HF severity was evaluated by analyzing LnPTH distribution across NYHA classes. As shown in Figure 2, a progressive increase in LnPTH concentrations was observed as functional status declined. Mean LnPTH levels rose significantly from 3.94 in NYHA Class I to 4.27 in Class III, indicating a direct association between PTH elevation and clinical disease progression.

### 3.3. Prognostic Performance of LnPTH and LnNT-proBNP

**Figure 3:**
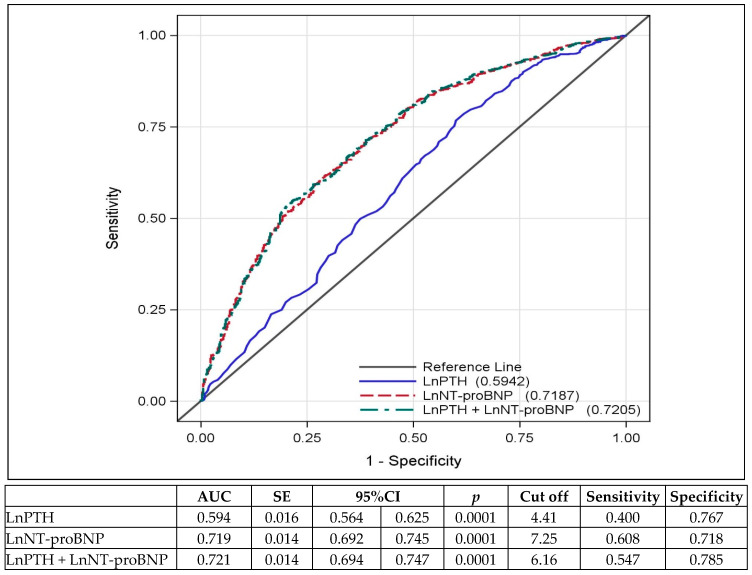
ROC Curve Analysis of LnPTH, LnNT-proBNP, and Combined Multi-Biomarker Model for Predicting Long-Term Mortality. Receiver operating characteristic curves showing the discriminative performance of LnPTH, LnNT-proBNP (LnBNP), and their combined multi-biomarker score for all-cause mortality.

ROC curve analysis was utilized to determine and compare the predictive capacity of the biomarkers for long-term all-cause mortality (Figure 3). Alone, LnPTH yielded an area under the curve (AUC) of 0.594 (95% CI: 0.564–0.625, *p* < 0.001) with an optimal prognostic cut-off of 4.41 (back-transformed to ≈82 pg/mL). LnNT-proBNP alone demonstrated an AUC of 0.719 (95% CI: 0.692–0.745, *p* < 0.001) with an optimal cut-off of 7.25 (back-transformed to ≈1400 pg/mL). Crucially, the multi-biomarker combination of LnNT-proBNP + LnPTH achieved the highest discriminative performance with an AUC of 0.7205 (95% CI: 0.694–0.747, *p* < 0.001) and an optimal combined cut-off score of 6.159.

To formally test model optimization, a pair-wise comparison of the ROC curves was performed (Table 2). The dual-biomarker model (LnNT-proBNP + LnPTH) significantly outperformed LnPTH alone (ΔAUC = 0.1263, χ^2^ = 63.59, *p* < 0.001). Similarly, LnNT-proBNP alone performed significantly better than LnPTH alone (ΔAUC = 0.1245, χ^2^ = 50.77, *p* < 0.001). Although the combined multi-biomarker model showed an absolute increase in AUC compared to LnNT-proBNP alone, this specific pair-wise increment did not reach statistical significance (ΔAUC = 0.0018, 95% CI: −0.0026 to 0.0061, χ^2^ = 0.644, *p* = 0.422), indicating that the raw discriminative curves overlap closely.

### 3.4. Long-Term Survival and Follow-Up Analysis

The median follow-up duration for the entire cohort was 36.0 months (Interquartile Range [IQR]: 18.0–67.2 months), with a mean follow-up of 42.2 ± 28.7 months and a maximum observation period of 118 months. During this period, 525 all-cause mortality events occurred.

**Figure 4:**
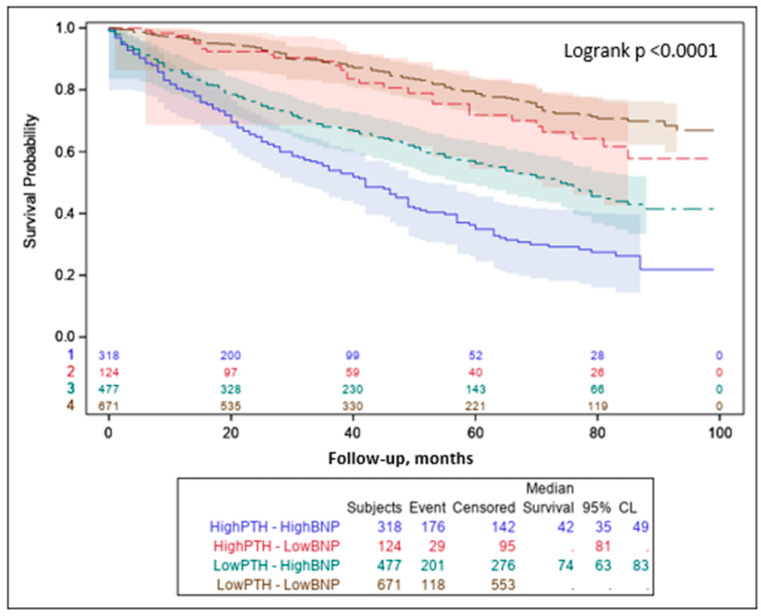
Kaplan–Meier Survival Analysis According to Combined PTH and NT-proBNP Stratification. Abbreviations: PTH, parathyroid hormone; NT-proBNP, N-terminal pro-brain natriuretic peptide; HFrEF, heart failure with reduced ejection fraction. LowPTH–LowBNP: LnPTH ≤ 4.41 + LnNT-proBNP ≤ 7.25; HighPTH–LowBNP: LnPTH > 4.41 + LnNT-proBNP ≤ 7.25; LowPTH–HighBNP: LnPTH ≤ 4.41 + LnNT-proBNP > 7.25; HighPTH–HighBNP: LnPTH > 4.41 + LnNT-proBNP > 7.25 Long-term cumulative survival probabilities stratified by dual-biomarker profiles. Patients were categorized into four distinct cohorts based on optimal prognostic cut-off values for log-transformed intact parathyroid hormone (LnPTH) and N-terminal pro-brain natriuretic peptide (LnNT-proBNP), as determined by Receiver Operating Characteristic (ROC) curve analysis. “.” indicates that median survival was not reached during the follow-up period.

Kaplan–Meier survival curves stratified by the ROC-derived cut-offs revealed distinct prognostic trajectories (Figure 4). The High PTH–High BNP group (LnPTH > 4.41 and LnNT-proBNP > 7.25) exhibited the worst clinical outcome, with a median survival of only 42 months (95% CI: 35–49) and 176 deaths. Conversely, the Low PTH–Low BNP group showed the highest cumulative survival (118 deaths among 671 subjects; Log-rank *p* < 0.0001).

### 3.5. Multivariable Predictors of All-Cause Mortality

**Figure 5:**
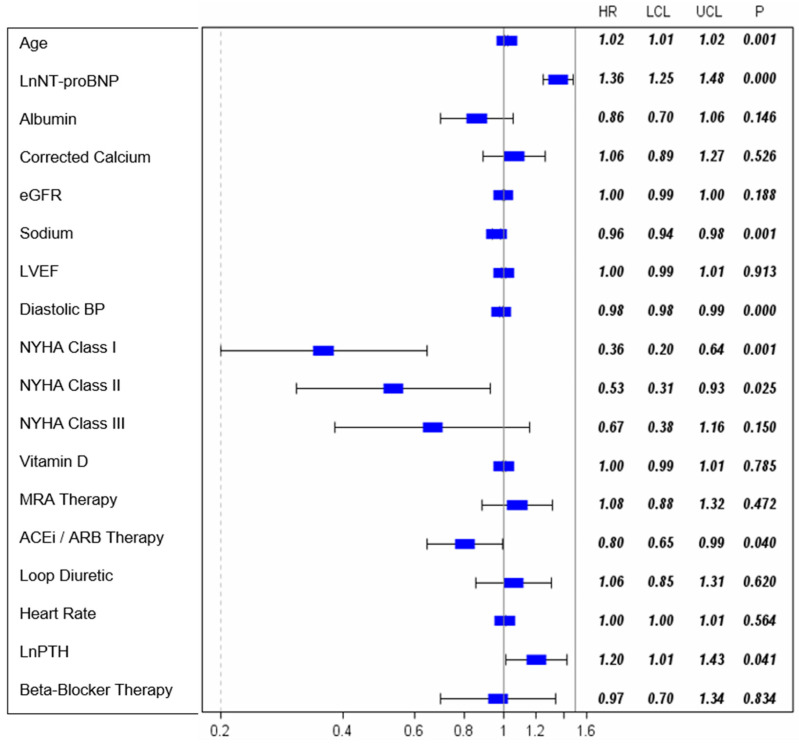
Forest plot of multivariable hazard ratios for key clinical and laboratory parameters. Forest plot showing hazard ratios (HR) with 95% confidence intervals for clinical subgroups and continuous variables. The vertical reference line indicates HR = 1. LnNT-proBNP refers to the natural logarithm of NT-proBNP, and LnPTH refers to the natural logarithm of PTH. Abbreviations: HR, hazard ratio; CI, confidence interval; NYHA, New York Heart Association functional class; BP, blood pressure; eGFR, estimated glomerular filtration rate; NT-proBNP, N-terminal pro–B-type natriuretic peptide; PTH, parathyroid hormone; ACEi, angiotensin-converting enzyme inhibitor; ARB, angiotensin receptor blocker; MRA, mineralocorticoid receptor antagonist.

To comprehensively evaluate independent risk factors and eliminate potential overfitting associated with automated stepwise elimination, a pre-specified, clinically driven forced-entry multivariable Cox proportional hazards model was implemented. All key demographics, echocardiographic parameters, neurohormonal/mineral markers, and heart failure medications were simultaneously entered into the model (Table 3, Figure 5).

After simultaneous adjustments, both log-transformed biomarkers retained highly significant independent associations with long-term all-cause mortality. For each single-unit increase in LnPTH, patients experienced a statistically significant 20.1% increase in the risk of death (Hazard Ratio [HR]: 1.201; 95% CI: 1.007–1.432, *p* = 0.0412). Similarly, each unit increase in LnNT-proBNP was associated with a 36.0% higher risk of mortality (HR: 1.360; 95% CI: 1.249–1.482, *p* < 0.0001). Other strong, independent predictors of all-cause mortality included advanced age (HR: 1.015 per year; *p* = 0.0006), serum sodium concentrations (HR: 0.959 per mEq/L; *p* = 0.0011), and diastolic blood pressure (HR: 0.983 mmHg; *p* < 0.0001). Consistent with therapeutic guidelines, the pre-specified entry of evidence-based medical treatments revealed that ACE Inhibitors/ARBs conferred significant survival protection (HR: 0.800; 95% CI: 0.647–0.990, *p* = 0.040).

Crucially, to verify whether the prognostic value of PTH was merely a surrogate for worsening renal function, eGFR was concurrently retained within the forced-entry model. Notably, while eGFR lost its independent statistical significance for mortality when adjusted simultaneously for the full panel (HR: 0.996, 95% CI: 0.991–1.002, *p* = 0.1879), LnPTH maintained a highly robust and independent predictor of death (*p* = 0.0412), underscoring a distinct myocardial or metabolic pathway separate from simple cardiorenal clearance.

To formally reconcile the discrepancy where the raw ROC curves overlap closely but LnPTH remains highly significant in multivariable modeling, advanced reclassification and likelihood diagnostics were performed. Formally, at the 36-month follow-up, the addition of LnPTH to the baseline conventional clinical model yielded an Integrated Discrimination Improvement (IDI) of 0.00107 (SE 0.00089, *p* = 0.2296) and a Net Reclassification Index (NRI) of 0.0448 (SE 0.0533, *p* = 0.4006), indicating that the absolute proportion of patients reclassified between risk strata did not change substantially on an absolute risk scale. However, the nested Likelihood Ratio (LR) test—which evaluates global model fit through reductions in model deviance—demonstrated that the addition of LnPTH to the reduced model significantly improved overall model performance (χ^2^ = 4.2419, *p* = 0.0394). Taken together, these findings indicate that although individual reclassification metrics showed modest changes, the inclusion of LnPTH provides meaningful improvement in global model fit and contributes independent prognostic information.

### 3.6. Subgroup and Interaction Analyses

To evaluate the clinical consistency of the prognostic value of lnPTH across predefined clinical strata, stratified multivariable Cox proportional hazards models and formal interaction examinations were executed (Table 4). The independent predictive power of lnPTH for long-term all-cause mortality was significantly modified by age group (p-interaction = 0.026) and baseline kidney function (p-interaction = 0.038).

In the age-stratified cohort, higher lnPTH levels independently predicted an increased risk of all-cause mortality among patients aged < 65 years (Adjusted HR: 1.402, 95% CI: 1.088–1.806, *p* = 0.009). Conversely, this relationship lost statistical significance in elderly participants aged ≥ 65 years (Adjusted HR: 0.966, 95% CI: 0.748–1.247, *p* = 0.788). A complementary pattern was observed when the population was stratified by renal efficiency; higher lnPTH levels were robust independent predictor of death among patients with preserved or only mildly reduced renal function (eGFR ≥ 60 mL/min/1.73 m^2^; Adjusted HR: 1.454, 95% CI: 1.129–1.873, *p* = 0.004). However, the independent risk association disappeared in those presenting with moderate-to-severe kidney impairment (eGFR < 60 mL/min/1.73 m^2^; Adjusted HR: 1.074, 95% CI: 0.837–1.379, *p* = 0.574). In contrast, the prognostic relative risk of lnPTH remained remarkably uniform regardless of the heart failure clinical etiology (p-interaction = 0.289). While the intra-stratum calculation showed a nominal independent significance within the non-ischemic patient subset (Adjusted HR: 1.332, 95% CI: 1.008–1.759, *p* = 0.044) and did not meet the traditional alpha threshold in the ischemic patient subgroup (Adjusted HR: 1.096, 95% CI: 0.879–1.367, *p* = 0.417), the high p-interaction value mathematically demonstrates that the overarching predictive trend of secondary hyperparathyroidism does not dynamically change between ischemic and non-ischemic heart failure etiologies.

## 4. Discussion

In this large, well-characterized cohort of patients with HFrEF (*n* = 1594), the principal finding of our study is that parathyroid hormone (PTH) traditionally viewed as a bone mineral regulator acts as a clinically relevant component of the cardiorenal–mineral axis and is independently associated with long-term all-cause mortality in HFrEF [16]. Beyond its prognostic significance, we observed a clear stepwise increase in LnPTH levels across worsening NYHA functional classes, indicating that PTH also reflects clinical severity in HFrEF. Together, these findings support the concept that neurohumoral activation in HFrEF is tightly coupled to disease progression and that contemporary biomarker strategies should integrate signals beyond myocardial stretch alone [16].

**Figure 6:**
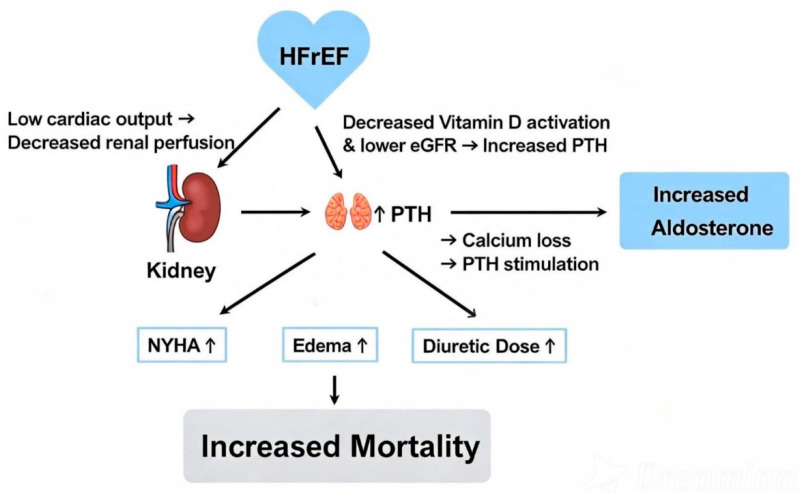
Central Illustration: Integrated Cardiorenal–Mineral Axis in HFrEF. A mechanistic overview depicting the interplay between reduced cardiac output, impaired renal perfusion, altered vitamin D metabolism, secondary hyperparathyroidism, aldosterone activation, and their convergence on adverse outcomes in HFrEF. Arrows indicate the pathophysiological pathway and the progression of clinical outcomes associated with increased parathyroid hormone (PTH) in patients with HFrEF. Abbreviations: HFrEF, heart failure with reduced ejection fraction; PTH, parathyroid hormone.

The integrated pathophysiological framework underlying these findings is summarized in the central illustration (Figure 6). This schematic highlights the interconnected cardiorenal–mineral axis linking reduced cardiac output, impaired renal perfusion, altered vitamin D metabolism, secondary hyperparathyroidism, and aldosterone activation, providing a mechanistic context for the observed association between elevated PTH and adverse outcomes.

A key observation was the progressive rise in LnPTH across NYHA classes, consistent with earlier studies reporting higher intact PTH concentrations in advanced HF and correlations with established markers of disease severity [14]. Mechanistically, chronic RAAS activation and diuretic exposure may promote secondary hyperparathyroidism through renal and gastrointestinal calcium wasting and impaired vitamin D activation, thereby sustaining PTH secretion over time [17]. This endocrine–renal–cardiac interplay has been emphasized in prior reviews describing PTH as part of a broader neurohormonal milieu in HF rather than an isolated mineral marker [18,19].

Although the discriminative ability of PTH alone was modest in ROC analysis, its clinical value became more apparent when interpreted within a multimarker framework. Natriuretic peptides remain the cornerstone biomarkers for diagnosis and prognostic assessment, reflecting myocardial stretch and hemodynamic load [20]. PTH, in contrast, conveys distinct biological information related to mineral metabolism, neurohormonal activation, and systemic remodeling, thereby complementing NT-proBNP-based risk stratification [21]. In line with this conceptual model, our Kaplan–Meier analyses demonstrated that the combined “high PTH–high NT-proBNP” phenotype identified the subgroup with the poorest survival, suggesting a biological synergy between endocrine–metabolic stress and myocardial stretch.

To further characterize the incremental prognostic contribution of PTH beyond established risk factors, we executed formal statistical metrics including the Likelihood Ratio (LR) test, Net Reclassification Index (NRI), and Integrated Discrimination Improvement (IDI). While the standalone ROC area under the curve for LnPTH was close to chance (AUC = 0.594) compared to LnNT-proBNP (AUC = 0.719), the dual biomarker combination of LnNT-proBNP + LnPTH marginally enhanced the discriminative metric to an AUC of 0.7205 (95% CI: 0.694–0.747, *p* < 0.001). Pair-wise comparison verified that while the combined parameter and natriuretic peptide models both significantly outperformed PTH alone (*p* < 0.0001), the absolute difference in AUC between the dual model and NT-proBNP did not achieve statistical significance (estimate: 0.0018, *p* = 0.4224). Similarly, the formal reclassification analyses indicated that the full model containing LnPTH yielded a modest, non-significant reclassification index (NRI = 0.0448%, *p* = 0.4006) and an integrated discrimination index of 0.00107% (*p* = 0.2296). However, utilizing nested model evaluation via the Likelihood Ratio test revealed that the addition of LnPTH to the reduced model significantly improved the global model fit (Deviance Chi-Square = 4.2419, df = 1, *p* = 0.0394). This underscores that although PTH does not remarkably expand the linear area under the receiver operating curve, it provides independent, non-redundant mathematical optimization to the overall risk stratification structure, confirming better data fit.

Our findings extend the existing literature in several important ways. While Altay et al. demonstrated an association between serum PTH levels and HF severity, their study did not evaluate long-term mortality or incremental prognostic value [14]. Similarly, although Gruson et al. [19] proposed a multimarker strategy incorporating PTH and natriuretic peptides, their cohort included a heterogeneous chronic HF population. In contrast, our study provides robust evidence within a large, etiologically homogeneous HFrEF cohort, demonstrating that PTH offers independent and incremental prognostic value beyond NT-proBNP. These results therefore provide a more definitive and clinically applicable risk stratification framework than previously reported.

The plausibility of PTH as more than a severity marker is supported by mechanistic evidence. Elevated PTH has been linked to cardiomyocyte hypertrophy, arrhythmogenicity, intracellular calcium overload, and a pro-oxidative/pro-fibrotic milieu that may accelerate adverse remodeling [8,21,22]. Experimental frameworks describing the “calcium paradox” systemic calcium depletion with intracellular calcium excess offer a coherent mechanistic bridge between secondary hyperparathyroidism and myocardial injury pathways [23].

Our final model incorporated an exhaustive, pre-specified panel adjusting for all clinically relevant baseline factors, laboratory parameters, and contemporary guideline- directed medical therapies (GDMTs), including beta-blockers, ACEi/ARBs, MRAs, and loop diuretics. Within this rigorous fully adjusted framework, LnPTH sustained its independent prognostic significance for all-cause mortality (HR: 1.201, 95% CI: 1.007–1.432, *p* = 0.0412), proving that its predictive capacity is completely autonomous from traditional markers like age, eGFR, left ventricular ejection fraction (LVEF), or baseline natriuretic peptides. To further refine the clinical utility of this biomarker, we conducted formal subgroup and interaction analyses, which unveiled highly nuanced, phenotype-specific prognostic patterns. Notably, the independent predictive power of LnPTH for long-term all-cause mortality was significantly modified by age (p interaction = 0.026) and baseline renal function (p interaction = 0.038). In stratum-specific evaluations, higher LnPTH levels emerged as a powerful, independent predictor of death specifically among younger patients aged < 65 years (Adjusted HR: 1.402, 95% CI: 1.088–1.806, *p* = 0.009) and those with preserved or only mildly reduced renal function (eGFR ≥ 60 mL/min/1.73 m^2^; Adjusted HR: 1.454, 95% CI: 1.129–1.873, *p* = 0.004). Conversely, this prognostic association lost statistical significance in elderly participants (≥65 years) and those with moderate-to-severe kidney impairment (eGFR < 60 mL/min/1.73 m^2^). From a pathophysiological standpoint, these findings are highly revealing. In advanced age and severe chronic kidney disease (CKD), circulating PTH frequently undergoes secondary shifts driven by fixed structural nephron loss, chronic phosphate retention, and generalized senescent metabolic changes, which may dilute its specific cardiotoxic signal. In sharp contrast, the robust risk acceleration observed in younger individuals and those with preserved eGFR suggests that in the absence of advanced renal failure, early parathyroid overactivation represents a highly active, uncompensated neurohumoral and metabolic stress driver rather than a passive bystander of kidney dysfunction. Furthermore, the prognostic relative risk of LnPTH remained remarkably uniform across heart failure etiologies (p interaction = 0.289), demonstrating that the detrimental effects of secondary hyperparathyroidism operate via generalized systemic myocardial pathways that transcend whether the primary HFrEF insult was ischemic or non-ischemic in origin. Furthermore, regarding therapeutic baseline profiles, a high proportion of our cohort was treated with contemporary heart failure therapies; however, the utilization of advanced neurohormonal modulators such as Angiotensin Receptor–Neprilysin Inhibitors (ARNIs) and Sodium–Glucose Cotransporter-2 inhibitors (SGLT2i) was relatively limited due to the historical inclusion window spanning back to 2014, which represents an important descriptive consideration since modern treatments can profoundly modify neurohormonal loops and mineral balances.

A clinically relevant observation in our dataset was the apparent therapeutic discordance among patients with elevated PTH, particularly regarding MRA use. Prior studies have described a bidirectional “aldosterone–PTH” cross-talk: aldosterone promotes urinary calcium loss and secondary hyperparathyroidism, while PTH may stimulate adrenal aldosterone synthesis, reinforcing a maladaptive endocrine loop [13,24,25]. In this context, lower MRA utilization among high-PTH patients, often those with lower eGFR and higher perceived hyperkalemia risk, may represent a missed opportunity to attenuate a biologically relevant pathway linked to fibrosis and remodeling [5]. Although hypothesis-generating, these findings align with calls for more individualized, pathway-informed HF management strategies. In addition to neurohormonal markers, serum sodium emerged as a highly robust independent predictor of survival in our fully adjusted Cox model (HR: 0.959, 95% CI: 0.935–0.983, *p* = 0.0011). Electrolyte imbalances, particularly hyponatremia, are tightly interwoven with advanced neurohormonal activation, heightened argipressin secretion, and aggressive loop diuretic regimes in fluid-overloaded heart failure states. This profound prognostic relevance of sodium shifts is thoroughly consistent with recent literature highlighting that intricate electrolyte metrics, such as the sodium-to-chloride ratio, serve as direct indices of cardiorenal tension, diuretic resistance, and mortality risk in heart failure cohorts, demanding precise clinical vigilance [26].

Our results also align with broader epidemiological evidence showing that PTH tracks cardiovascular vulnerability even in the absence of overt mineral disturbances. Elevated PTH predicted cardiovascular mortality in community cohorts, including individuals with values within the normal reference range [10]. A meta-analysis further demonstrated that higher PTH levels were associated with increased risk of incident HF in the general population, supporting its biological relevance across the HF continuum [27]. At the same time, prior HF studies have reported heterogeneity regarding whether PTH retains independent prognostic value after adjustment for congestion and renal indices, suggesting that patient phenotype and covariate structure may influence effect sizes [15]. The fact that PTH remained independently associated with mortality in our large HFrEF cohort strengthens the argument that PTH contributes additive prognostic information, particularly when evaluated alongside natriuretic peptides [14,24].

Crucially, the persistent elevation of PTH in HFrEF must be contextualized within the broader framework of chronic systemic inflammation, which represents a fundamental coexisting driver of adverse myocardial remodeling, endothelial dysfunction, and progressive cachexia in heart failure [28]. Furthermore, this phenomenon is increasingly recognized as an integral component of the cardiovascular–kidney–metabolic (CKM) syndrome. Metabolic disturbances, such as insulin resistance, visceral adiposity, and metabolic dysfunction-associated steatotic liver disease (MASLD), are known to trigger early, subclinical myocardial mechanical impairment, often detectable as reduced left ventricular global longitudinal strain (LV-GLS) via advanced speckle-tracking echocardiography long before an explicit decline in LVEF becomes manifest [29,30]. Given that elevated circulating PTH is frequently encountered in patients combining metabolic stress, chronic kidney disease, and high inflammatory loads, PTH may effectively serve as an aggregated biomarker reflecting the cumulative systemic metabolic, renal, and mechanical burden imposed on the myocardium.

From a clinical perspective, these findings support the consideration of PTH as a complementary biomarker to refine long-term risk stratification in HFrEF. Integrating signals from distinct biological pathways aligns with broader multimarker strategies, where combining natriuretic peptides with additional indices improves prognostic accuracy over single-marker approaches. To bridge these scientific findings with real-world clinical practice, we propose a practical clinical diagnostic and management algorithm incorporating PTH evaluation. In patients presenting with HFrEF, a comprehensive baseline mineral and biomarker panel—comprising NT-proBNP, baseline intact PTH, serum calcium, phosphate, 25(OH)D, and eGFR—should be performed. Patients demonstrating dual elevation (LnPTH > 4.41 or ~82 pg/mL, combined with LnNT- proBNP > 7.25 or ~1400 pg/mL) should be risk-stratified into the highest vulnerability tier, requiring intensive clinical surveillance and rapid up-titration of core guideline-directed medical therapies. Furthermore, in these high-PTH individuals, specific attention must be paid to excluding secondary hyperparathyroidism secondary to correctable vitamin D deficiency (by supplementing 25(OH)D to optimize mineral homeostasis), minimizing excessive loop diuretic congestion-wasting, and strictly prioritizing the initiation or optimization of MRAs to block the maladaptive aldosterone–PTH feed-forward loop, provided renal parameters permit. Future prospective studies are warranted to (i) validate the incremental prognostic value of PTH using contemporary metrics such as net reclassification improvement, (ii) determine whether serial PTH measurements provide dynamic prognostic information, and (iii) evaluate whether therapeutic modulation of the aldosterone–mineral axis, including optimized MRA use, can influence PTH trajectories and clinical outcomes.

## 5. Conclusions

In conclusion, our study demonstrates that PTH is independently associated with clinical severity and long-term all-cause mortality in a large, well-characterized cohort of patients with HFrEF. Elevated LnPTH levels track tightly with worsening NYHA functional classes, reflecting its role as an integrated marker within the systemic cardiorenal–mineral axis. Importantly, although the standalone discriminative capacity of PTH is modest, nested model evaluations confirm that it provides independent, non-redundant optimization to traditional natriuretic peptide-based risk stratification systems. Furthermore, our findings highlight a crucial therapeutic discordance, where high-vulnerability patients with elevated PTH and complex mineral stress are less likely to receive mineralocorticoid receptor antagonists (MRAs). When contextualized within the broader cardiovascular–kidney–metabolic (CKM) syndrome and chronic inflammation, PTH emerges as a comprehensive surrogate for cumulative metabolic and hemodynamic load. These results support the implementation of a multimarker clinical algorithm to refine long-term risk assessment and encourage pathway-informed, individualized therapeutic strategies in HFrEF.

## 6. Limitations

Our study is subject to several limitations. First, its retrospective, single-center design limits the ability to establish causality and may affect the generalizability of the results to broader populations. Second, out of 2185 screened individuals, a significant proportion (*n* = 420, approximately 20%) were excluded due to missing baseline PTH or 25(OH)D values, which introduces potential selection bias. Nonetheless, an exhaustive comparative analysis between the main study cohort and the excluded cohort (Supplementary Table S1) demonstrated that both groups were closely matched regarding major clinical and hemodynamic characteristics (including age, LVEF, and NYHA class), although the excluded cohort exhibited higher baseline NT-proBNP, indicating that they represented a highly vulnerable subset. Third, the reliance on a single baseline measurement prevents the assessment of longitudinal PTH fluctuations and dynamic therapeutic responses, highlighting the essential need for future prospective studies incorporating serial biomarker monitoring. Fourth, fibroblast growth factor 23 (FGF-23), a crucial regulator of the phosphate–mineral axis tightly intertwined with PTH biology, was not measured in our dataset. Fifth, our optimal prognostic cut-off of LnPTH = 4.41 (~82 pg/mL) was derived strictly from the current cohort using ROC analysis and has not been validated on an external independent population, which exposes the threshold to potential overfitting and requires caution before direct general application. Sixth, the primary endpoint was all-cause mortality; due to data constraints, we were unable to adjudicate or analyze specific cardiovascular mortality versus non-cardiac causes, which is highly relevant given that HFrEF patients harbor significant competing risks. Finally, the modest standalone discriminative power of PTH suggests that it should be integrated into comprehensive risk frameworks rather than utilized as a primary prognostic tool. 

## Figures and Tables

**Table 1:** Baseline Demographic, Clinical, Laboratory Characteristics, and Medications of Patients.

Variable	Total (N = 1594)	PTH ≤ 82 (N = 1150)	PTH > 82 (N = 444)	*p* Value
Demographics				
Sex (Male), n (%)	1168 (73.3%)	76.5%	64.9%	<0.001
Age, (years)	62.1 ± 12.8	60.7 ± 12.8	65.8 ± 12.0	<0.001
Heart Failure Duration, (months)	12 (3.0–48)	12 (3–36)	24 (4–72)	<0.001
Etiology of HF (Ischemic), n (%)	885 (55%)	643 (56%)	242 (55%)	0.612
Clinical and Echocardiographic Parameters				
NYHA				<0.001
NYHA I, n (%)	428 (26.9%)	352 (30.6%)	76 (17.1%)	
NYHA II, n (%)	847 (53.1%)	611 (53.1%)	236 (53.2%)	
NYHA III, n (%)	303 (19.0%)	179 (15.6%)	124 (27.9%)	
NYHA IV, n (%)	16 (1.0%)	8 (0.7%)	8 (1.8%)	
LV EF, %	29.5 ± 8.1	29.7 ± 8.0	29.0 ± 8.2	0.123
LV EDD, mm	57.8 ± 7.7	57.7 ± 7.8	58.1 ± 7.4	0.249
LV ESD, mm	46.9± 8.9	46.7 ± 8.9	47.3 ± 8.8	0.261
Systolic BP, mmHg	116.8 ± 20.0	116.8 ± 19.7	116.6 ± 21.0	0.894
Diastolic BP, mmHg	70.0 ± 12.9	69.9 ± 12.5	70.4 ± 14.1	0.509
Heart Rate, (bpm)	77.4 ± 17.4	75.9 ± 16.4	81.4 ± 19.3	<0.001
Comorbidities, n (%)				
Hypertension	908 (57.0%)	630 (54.7%)	278 (62%)	0.005
Diabetes Mellitus	733 (46.0%)	537 (46%)	196 (44%)	0.36
Hyperlipidemia	923 (57.9%)	682 (59.3%)	241 (54.3%)	0.07
Atrial Fibrillation	271 (17%)	132 (11%)	139 (31%)	<0.001
Laboratory Data				
Sodium, mEq/L	138.8 ± 3.3	138.7 ± 3.2	139.1 ±3.5	0.019
Potassium, mEq/L	4.59 ± 0.49	4.6 ± 0.46	4.5 ± 0.5	0.059
Magnesium, mg/dL	2.0 ± 0.2	2.0 ±0.2	2.1 ± 0.2	0.001
Calcium, mg/dL	9.6 ± 0.6	9.64 ±0.55	9.41 ± 0.6	<0.001
Corrected Calcium, mg/dL	9.4 ± 0.5	9.44 ± 0.5	9.3 ± 0.56	<0.001
ALT, U/L	18(13–25)	18 (13–26)	17 (13–25)	0.555
AST, U/L	20 (16–24)	19 (16–24)	20 (16–25)	0.031
eGFR, mL/min/1.73 m^2^	68 (53–82)	72 (57–85)	58 (46–73)	<0.001
Creatinine, mg/dL	1.1 (1.0–1.3)	1.1 (0.9–1.2)	1.2 (1.0–1.4)	<0.001
BUN, mg/dL	19.0 (15.0–25.0)	18 (14–23)	22 (17–29)	<0.001
Hemoglobin, g/dL	13.5 ± 1.9	13.6 ±1.9	13.0 ± 1.9	0.001
Glucose, mg/dL	104 (90–134)	105 (90–137)	104 (91–129)	0.08
NT-proBNP, ng/L	1403 (474–3370)	992 (381–2579)	2826 (1140–5734)	<0.001
hs-Troponin T, ng/L	12 (10–25)	11 (10–21)	16 (10–33)	<0.001
PTH, ng/L	59 (42–87)			
Vitamin D, µg/L	19 (13.1–27)	20.3(14.3–28.0)	15.6 (10.5–23.0)	<0.001
Total Cholesterol, mg/dL	170.7 ± 49.5	172.4 ± 50.3	166.3 ± 47.0	0.022
HbA1c, %	6.7± 1.5	6.8 ± 1.6	6.7 ± 1.4	0.163
Albumin, g/L	4.2 ± 0.5	4.3 ±0.4	4.1 ± 0.5	<0.001
Medication, n (%)				
ACE Inh./ARB	1268 (80%)	946(82%)	277 (73%)	<0.001
ARNI	117 (7.3%)	75 (6.5%)	42 (9.5%)	0.146
Beta-blockers	1501 (94.2%)	1090 (95%)	411 (93%)	0.091
Calcium Channel Blockers	133 (8.3%)	81 (7%)	52 (12%)	0.06
MRA, n (%)	1158(72.6%)	873 (76%)	285 (64%)	<0.001
Loop Diuretics	1018 (63.9%)	667 (58%)	351 (79%)	<0.001
Loop Diuretic Dose, mg	40 (0–40)	20 (0–40)	40 (20–40)	<0.001
Digoxin	132 (8.3%)	85 (7.4%)	47 ((11%)	0.038
Ivabradine	158 (9.9%)	114 (9.9%)	44 (9.9%)	0.999
Statins	808 (50.7%)	615 (54%54%)	193 (43%)	<0.001
Acetylsalicylic acid	932 (58.5%)	707 (62%)	225 (51%)	<0.001
Oral Anticoagulants	369 (23%)	210 (18%)	159 (36%)	<0.001
SGLT2 Inhibitors	302 (18.9%)	235 (20%)	67 (15%)	0.045

Abbreviations: ACE, angiotensin-converting enzyme; ARB, angiotensin receptor blocker; ARNI, angiotensin receptor–neprilysin inhibitor; AST, aspartate aminotransferase; ALT, alanine aminotransferase; bpm, beats per minute; BP, blood pressure; BUN, blood urea nitrogen; eGFR, estimated glomerular filtration rate; HbA1c, glycated hemoglobin; HF, heart failure; hs-Troponin T, high-sensitivity troponin T; LV EDD, left ventricular end-diastolic diameter; LV EF, left ventricular ejection fraction; LV ESD, left ventricular end-systolic diameter; MRA, mineralocorticoid receptor antagonist; NT-proBNP, N-terminal pro-B-type natriuretic peptide; NYHA, New York Heart Association; PTH, parathyroid hormone; SGLT2, sodium–glucose cotransporter-2.

**Table 2:** Pair-wise comparisons of area under the receiver operating characteristic curve (AUC) for biomarkers.

Contrast	Estimate	Standard Error	95% CI	Chi-Square	*p*-Value
(LnNT-proBNP + LnPTH) − LnPTH	0.126	0.016	(0.095–0.157)	63.59	<0.0001
LnNT-proBNP − LnPTH	0.125	0.018	(0.090–0.159)	50.77	<0.0001
(LnNT-proBNP + LnPTH) − LnNT-proBNP	0.002	0.002	(−0.003–0.006)	0.64	0.4224

**Table 3:** Multivariable Cox Proportional Hazards Regression Analysis for Predicting All-Cause Mortality in Patients with HFrEF.

Parameter	Coefficient (*β*)	Standard Error	Chi-Square (χ^2^)	*p*-Value	Hazard Ratio (95% CI)
Age	0.0149	0.0043	11.721	0.0006	1.015 (1.006–1.024)
LnNT-proBNP	0.3077	0.0437	49.521	<0.0001	1.360 (1.249–1.482)
Albumin	−0.1549	0.1064	2.119	0.1455	0.856 (0.695–1.055)
CorrectedCa	0.0574	0.0904	0.403	0.5256	1.059 (0.887–1.265)
eGFR	−0.0038	0.0029	1.734	0.1879	0.996 (0.991–1.002)
Sodium	−0.0418	0.0128	10.711	0.0011	0.959 (0.935–0.983)
LVEF	−0.00064	0.0058	0.012	0.9125	0.999 (0.988–1.011)
Diastolic BP	−0.0169	0.0039	18.717	<0.0001	0.983 (0.976–0.991)
NYHA Class I	−1.0246	0.2979	11.824	0.0006	0.359 (0.200–0.644)
NYHA Class II	−0.6297	0.2814	5.006	0.0253	0.533 (0.307–0.925)
NYHA Class III	−0.4066	0.2826	2.069	0.1503	0.666 (0.383–1.159)
Vitamin D	−0.0011	0.0039	0.074	0.7847	0.999 (0.991–1.007)
MRA Therapy	0.0735	0.1021	0.517	0.4720	1.076 (0.881–1.315)
ACEi/ARB Therapy	−0.2227	0.1084	4.216	0.0400	0.800 (0.647–0.990)
Loop Diuretic	0.0541	0.1090	0.246	0.6200	1.056 (0.852–1.307)
Heart Rate	0.0015	0.0026	0.333	0.5636	1.002 (0.996–1.007)
LnPTH	0.1832	0.0897	4.167	0.0412	1.201 (1.007–1.432)
Beta-Blocker Therapy	−0.0351	0.1668	0.044	0.8336	0.966 (0.696–1.339)

Abbreviations: ACEi, angiotensin-converting enzyme inhibitor; ARB, angiotensin receptor blocker; BP, blood pressure; CI, confidence interval; eGFR, estimated glomerular filtration rate; HFrEF, heart failure with reduced ejection fraction; LnNT-proBNP, log-transformed N-terminal pro-B-type natriuretic peptide; LnPTH, log-transformed parathyroid hormone; LVEF, left ventricular ejection fraction; MRA, mineralocorticoid receptor antagonist; NYHA, New York Heart Association. Note: *β* coefficient represents the log hazard ratio. NYHA Class IV was utilized as the reference category for the New York Heart Association functional classification. A *p*-value of <0.05 was considered statistically significant.

**Table 4:** Multivariable Cox Proportional Hazards Models and Interaction Analysis for Predictors of All-Cause Mortality Across Clinical Subgroups.

Subgroup Factors	N	Deaths (Events)	Adjusted Hazard Ratio (95% CI)	Subgroup *p*-Value	p-Interaction
Age					0.026
<65 years	931	232	1.402 (1.088–1.806)	0.009	
≥65 years	663	292	0.966 (0.748–1.247)	0.788	
eGFR					0.038
≥60 mL/min/1.73 m^2^	1004	264	1.454 (1.129–1.873)	0.004	
<60 mL/min/1.73 m^2^	590	260	1.074 (0.837–1.379)	0.574	
Heart Failure Etiology					0.289
Non-Ischemic	709	220	1.332 (1.008–1.759)	0.044	
Ischemic	885	304	1.096 (0.879–1.367)	0.417	

Note: Individual subgroup hazard ratios (HRs) were calculated via stratified multivariable Cox regression models. p-interaction values represent the significance of the interaction term (lnPTH × Subgroup Variable) entered into the non-stratified fully adjusted model. All models are simultaneously adjusted for age, sex, lnNT-proBNP, albumin, corrected calcium, eGFR, sodium, LVEF, diastolic blood pressure, NYHA functional class, vitamin D levels, heart rate, and guideline-directed medical therapies (ACEi/ARB, MRA, beta-blockers, and diuretics).

## Data Availability

The data supporting the findings of this study are available from the corresponding author upon reasonable request. Due to institutional privacy regulations, raw patient-level data cannot be publicly shared.

## Supplementary Materials

The following supporting information can be downloaded at: https://www.mdpi.com/article/10.3390/jcm15134859/s1, Table S1. Baseline Characteristics and Clinical Profiles of the Main Study Cohort vs. Excluded Patients Due to Missing Biomarker Data.
