# Supplementary material for: Prognostic Value of Parathyroid Hormone in Heart Failure with Reduced Ejection Fraction
Source: J Clin Med. 2026 Jun 23;15(13):4859. doi: 10.3390/jcm15134859 (PMC13361963; doi:10.3390/jcm15134859)
Supplement: Supplementary file 1 [file jcm-15-04859-s001.zip › jcm-4366631-supplementary.pdf]

Supplementary Table S1. Baseline Characteristics and Clinical Profiles of the Main Study Cohort vs. Excluded Patients Due to Missing Biomarker Data.

Baseline Characteristics	Excluded Cohort (Missing PTH/VitD) (n = 420)	Main Analysis Cohort (Full Data) (n = 1,594)	p-value
**Demographics & Clinical History**			
Age (years), Mean ± SD	63.71 ± 13.28	62.15 ± 12.78	0.082
Female Gender, % (n)	28.5% (120/420)	26.8% (427/1594)	0.474
Duration of HF (months), Mean ± SD	36.92 ± 50.35	35.64 ± 53.32	0.655
HF Etiology (Ischemic), % (n)	56.1% (236/420)	55.0% (877/1594)	0.940
Hypertension, % (n)	50.8% (213/420)	57.0% (909/1594)	0.018
Diabetes Mellitus, % (n)	39.2% (165/420)	46.1% (735/1594)	0.009
Smoking History, %	Median/IQR Reported	Median/IQR Reported	<0.001
Body Mass Index (kg/m²), Mean ± SD	26.92 ± 5.59	27.41 ± 4.95	0.042
**Hemodynamic Parameters & Severity**			
Systolic Blood Pressure (mmHg)	115.17 ± 19.98	116.67 ± 20.24	0.181
Diastolik Blood Pressure (mmHg)	67.89 ± 12.53	69.99 ± 13.04	0.062
Heart Rate (bpm), Mean ± SD	78.11 ± 12.40	77.44 ± 11.90	0.315
Left Ventricular Ejection Fraction	29.10 ± 6.20	29.49 ± 5.80	0.238

(%)			
NYHA Functional Class, Mean ± SD	1.93 ± 0.80	1.94 ± 0.71	0.514
HF Hospitalization in Past Year, Mean	0.51	0.37	0.012
**Guideline-Directed Medical Therapies**			
ACEi / ARB Therapy, %	73.3%	68.4%	0.196
ARNI (Sacubitril/Valsartan), %	2.7%	7.4%	<0.001
Beta-Blockers, %	95.3%	93.9%	0.358
Mineralocorticoid Receptor Antagonists	73.5%	70.4%	0.794
Loop Diuretics, %	70.8%	63.5%	0.006
SGLT2 Inhibitors, %	Small usage	Higher usage	0.110
**Laboratory Parameters**			
Hemoglobin (g/dL), Mean ± SD*	12.95 ± 1.99	13.42 ± 1.85	0.054
Serum Glucose (mg/dL), Mean ± SD	128.83 ± 64.68	124.17 ± 59.56	0.416
eGFR (CKD-EPI, mL/min/1.73m^2)	66.28 ± 19.65	68.24 ± 20.49	0.085
Serum Sodium (mmol/L), Mean ± SD	137.35 ± 3.90	138.83 ± 3.31	<0.001
Corrected Calcium (mg/dL), Mean ± SD	9.21 ± 0.62	9.69 ± 3.11	<0.001
Serum Albumin (g/dL), Mean ± SD	3.99 ± 1.73	4.42 ± 2.84	<0.001

Serum Uric Acid (mg/dL), Mean ± SD	6.99 ± 2.07	7.05 ± 9.38	0.207
NT-proBNP (pg/mL), Mean ± SD	3844.81 ± 5463.56	3172.07 ± 5253.28	0.002
Vitamin D (ng/mL), Mean ± SD	20.15 ± 8.40	21.34 ± 9.10	0.230

*Note: Categorical variables are expressed as percentages and counts. Continuous variables are expressed as Mean ± Standard Deviation or Median (Interquartile Range) depending on data distribution normality. The 'Excluded Cohort' denotes patients excluded due to missing baseline intact parathyroid hormone (PTH) or 25-hydroxyvitamin D [25(OH)D] values. *The anomalous physiological standard deviation reported for hemoglobin in raw data was cross-referenced and cleaned using filtered laboratory range limits. Statistically significant p-values (<0.05) are indicated in bold.*

Abbreviations: ACEi: Angiotensin-Converting Enzyme Inhibitor; AF: Atrial Fibrillation; ARB: Angiotensin II Receptor Blocker; ARNI: Angiotensin Receptor-Neprilysin Inhibitor; BMI: Body Mass Index; Ca: Corrected Calcium; CKD-EPI: Chronic Kidney Disease Epidemiology Collaboration; eGFR: Estimated Glomerular Filtration Rate; HF: Heart Failure; HR: Heart Rate; IQR: Interquartile Range; LVH: Left Ventricular Hypertrophy; MRA: Mineralocorticoid Receptor Antagonist; Na: Sodium; NT-proBNP: N-terminal pro-B-type Natriuretic Peptide; NYHA: New York Heart Association; SD: Standard Deviation; SGLT2: Sodium-Glucose Cotransporter-2.
